# Advances in Pharmacotherapy and Physiotherapy for Dry Eye Disease: Molecular Mechanisms and Future Directions—A Narrative Literature Review

**DOI:** 10.3390/ijms27094024

**Published:** 2026-04-30

**Authors:** Jiaxiang Liu, Haina Zheng, Jiashu Shi, Miaomiao Hao, Qin Yang, Hongdou Luo, Xu Zhang

**Affiliations:** 1 Affiliated Eye Hospital of Nanchang University, Nanchang University School of Ophthalmology & Optometry, Nanchang 330006, China18870803259@163.com (H.Z.); haomm2003@163.com (M.H.); qinyang.quentin@gmail.com (Q.Y.); 15270995219@163.com (H.L.); 2 Jiangxi Research Institute of Ophthalmology & Visual Science, Jiangxi Provincial Key Laboratory for Ophthalmology, Nanchang 330006, China; 3 Queen Mary College, Nanchang University, Nanchang 330006, China; jiashu_shi@163.com

**Keywords:** dry eye disease, treatment, nanotechnology, physiotherapy, lifestyle

## Abstract

Dry eye disease, a multifactorial and symptomatic disease characterized by tear film instability and ocular surface dysfunction, has emerged as an increasingly pressing global health concern—particularly against the backdrop of increasing digital device usage and the widespread application of virtual learning. Traditional pharmacotherapies, such as artificial tears, yield only transient symptomatic relief. Compared with pharmacological agents, surgical treatments are further restricted in clinical application, primarily because of their invasiveness, technical complexity, postoperative complications, and high costs. Consequently, the development of novel therapeutic strategies has emerged as imperative. This review summarizes advances in pharmacotherapy, including nanomedicine and biological agents, as well as emerging physiotherapies, such as photobiomodulation, thermal pulsation, and neurostimulation. These innovative therapeutic approaches address the partial limitations of conventional treatments through three main molecular mechanisms: improved drug delivery, multitargeted pharmacology, and enhanced biocompatibility. Nevertheless, the clinical translation of many innovative therapies requires large-scale clinical trials to validate clinical efficacy, optimize dosage regimens, and assess long-term safety profiles. In the future, integrating lifestyle modifications, effective clinician–patient communication, and patient-centric diagnostic approaches will facilitate the establishment of therapeutic alliances and support the success of precision medicine.

## 1. Introduction

Dry eye disease (DED) is a multifactorial and symptomatic disease characterized by disrupted homeostasis of the tear film and/or ocular surface and is often accompanied by tear film instability, hyperosmolarity, ocular surface inflammation, epithelial injury, and/or neurosensory dysfunction [1]. Given the absence of universally accepted diagnostic criteria for DED, global prevalence estimates currently vary widely—ranging from 5% to 50% across different studies [2,3]. In particular, the estimated prevalence of DED increased to 61.0% globally and 56.7% in Asia during the COVID-19 pandemic, markedly higher than the levels prior to the pandemic, possibly due to the proliferation of remote work and online education [4].

The classification of DED has expanded beyond the traditional dichotomy of aqueous tear-deficient and evaporative DED [5]. In accordance with the recent TFOS DEWS III, dry eye is etiologically subclassified into tear film deficiencies (including lipid, aqueous, and mucin/glycocalyx deficiencies), eyelid-related anomalies (involving blink dynamics/lid closure and the lid margin), and ocular surface abnormalities (including anatomical misalignment, neural dysfunction, epithelial damage, and primary inflammation/oxidative stress) [1]. The prevalent clinical manifestation of DED is tear film instability, which is mediated through three main pathological mechanisms: diminished tear secretion, rapid tear film breakup, and excessive evaporation [6]. Thus, the main objective of DED treatment is to restore tear film homeostasis by disrupting these pathological processes. First-line treatment involves the administration of artificial tears and anti-inflammatory drugs [7]. Nevertheless, these traditional one-size-fits-all therapeutic remedies have demonstrated limited and varying clinical efficacy in different populations, mainly because of the complex interaction of etiologies. The etiologies of DED are constitutively multiple, overlapping and interrelated [8]. For instance, tear film instability and ocular surface inflammatory injury are impacted by neurosensory dysfunction, androgen deficiency, and impaired mucin secretion [8]. The limitations of conventional approaches underscore the urgent need for therapies targeting multiple pathogenic factors. Therefore, in this review, the major existing DED therapies are evaluated, and the feasibility of emerging therapeutic strategies is summarized.

## 2. Methodology

We searched the PubMed, Scopus and Web of Science databases using different combinations of the following keywords: “dry eye treatment”, “nanotechnology”, “light therapy”, “neurostimulation”, “physiotherapy”, “lifestyle”, etc. Our inclusion criteria were as follows: (1) written in English and (2) published in peer-reviewed journals; furthermore, we (3) prioritized studies published between 2020 and 2026 and (4) prioritized the following study types: consensus reports, randomized clinical trials, and pivotal translational studies.

## 3. Pathophysiology

**Figure 1:**
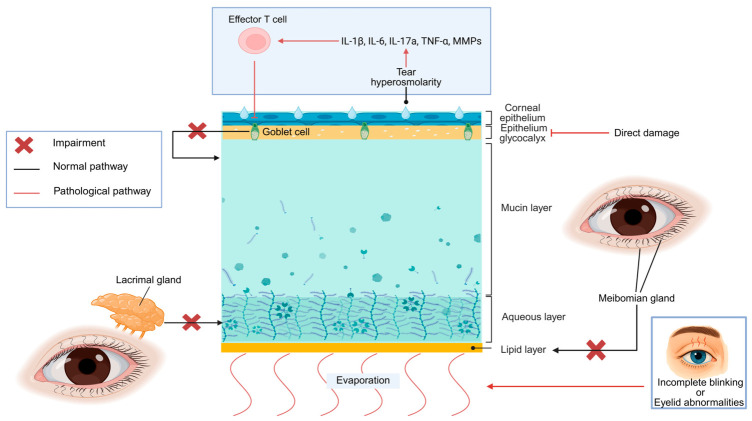
Pathophysiology of DED. The tear film consists of mucin, aqueous, and lipid layers, which are secreted by goblet cells, lacrimal glands, and meibomian glands, respectively. The pathophysiology of aqueous-deficient dry eye involves lacrimal gland dysfunction and ocular surface inflammation. Under the stress of tear hyperosmolarity, the release of inflammatory mediators leads to the activation of effector T cells, eventually resulting in goblet cell apoptosis and subsequent mucin deficiency. Concurrently, the impaired lacrimal gland compromises the secretion of the aqueous layer. Evaporative dry eye is caused primarily by MGD, incomplete blinking, eyelid abnormalities and corneal epithelial glycocalyx impairment. MGD reduces lipid secretion. Incomplete blinking or eyelid abnormalities promote excessive evaporation. Glycocalyx impairment exacerbates tear film instability.

The ocular surface comprises three anatomical structures: the cornea, the conjunctiva, and their protective coat—the tear film. The tear film consists of mucin, aqueous, and lipid layers (from inner to outer), which are synthesized by goblet cells, lacrimal glands and meibomian glands, respectively (Figure 1). Trigeminal sensory neurons, arising from the cornea or nasal mucosa, stimulate lacrimal gland secretion to maintain tear synthesis [9].

The pathophysiology of DED is multifactorial and centers on tear film instability. Dry eye arises from disturbed homeostasis of the lacrimal functional unit. This integrated system consists of ocular surface and adnexal structures, including the eyelids, lacrimal glands, and meibomian glands, as well as related sensory and motor neural pathways [8]. Two prevalent characteristics of DED are tear film instability and ocular surface inflammation. Any component in the lacrimal functional unit can lead to tear film instability [6].

Tear hyperosmolarity triggers ocular surface inflammation, characterized by the release of inflammatory mediators such as interleukin (IL)-1β [10], IL-6 [10], IL-17a [11], tumor necrosis factor-α (TNF-α) [12] and matrix metalloproteinases [13] (Figure 1). Under hyperosmolar stress, epithelial cells undergo desquamation, thereby exposing the underlying immature cells. The hydrophobicity of these cells directly destabilizes the tear film. The juxtaposition of these hydrophobic patches with hydrophilic cells creates a surface tension differential, prompting tear film breakup and evaporation within seconds [14]. This rapid evaporation markedly increases local osmolarity, establishing a vicious cycle of ocular surface injury, which is further amplified by inflammatory T cells [14]. Additionally, ocular surface inflammation compromises trigeminal neural circuits, which control the blink rate, tear secretion, and corneal sensitivity [15]. In turn, abnormal neurological transmission impairs tear secretion and blink integrity, resulting in another vicious cycle [15].

The two traditional subtypes of DED (aqueous-deficient and evaporative) have distinct pathological mechanisms (Figure 1). Aqueous-deficient dry eye is characterized by prominent inflammatory responses and lacrimal gland dysfunction [16]. Evaporative dry eye is attributable mainly to meibomian gland dysfunction (MGD), leading to impairment of the lipid layer [17]. Additional contributing factors include incomplete blinking—often associated with prolonged screen use—and direct damage to the corneal epithelial glycocalyx [18]. This mechanistic difference between subtypes underscores the necessity for subtype-specific diagnostic and therapeutic strategies: the management of aqueous-deficient dry eye should target inflammation and lacrimal function restoration, whereas evaporative dry eye therapy should focus on MGD management and glycocalyx repair [14].

Lacritin, a glycoprotein in the tear film, is involved in tear retention and antimicrobial defense along with other glycoproteins [19,20]. In-depth profiling of the tear fluid glycoproteome revealed the diversity of lacritin glycosylation patterns and splice variants [19]. The processed C-terminus of lacritin, which is deficient or absent in the tears of some dry eye patients, serves as a natural slow-release factor that can delay tear film collapse [21]. Moreover, lacritin acts as an inhibitor of multiple bacterial transporters, primarily through endogenous cleavage of the N-104 fragment, indicating its bactericidal activity [22]. The protective mechanisms of lacritin, coupled with its deficient state in DED individuals, suggest that lacritin is a promising candidate whose clinical efficacy still needs to be confirmed.

## 4. Treatment

### 4.1. Conventional Pharmacotherapy

#### 4.1.1. Tear Substitutes

**Figure 2:**
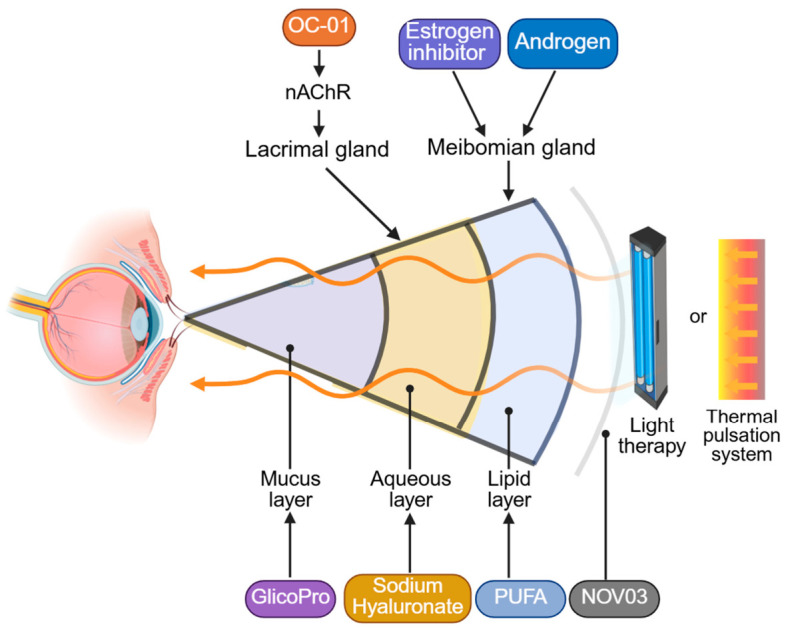
Main pathophysiology-based therapies for DED. Pharmacotherapies: OC-01 potentiates lacrimal gland secretion by activating nAchR. Estrogen inhibitors and androgens stimulate lipid secretion from the meibomian glands. The tear film supplements GlicoPro, sodium hyaluronate, and PUFA target the mucus, aqueous, and lipid layers, respectively. NOV03 assembles into a protective monolayer at the air–tear interface, thereby diminishing tear evaporation. Photobiomodulation therapies employ light pulses at various frequencies to stimulate lipid secretion by the meibomian glands. The thermal pulsation system employs a targeted temperature to soften solidified meibum, alleviating obstructions in the meibomian gland ducts.

Tear substitutes compensate for deficiencies in specific tear film components—the aqueous, mucin, and lipid layers. The main pathophysiology-based therapies for DED are summarized in Figure 2.

##### Artificial Tears

The first-line treatment of DED is artificial tear replacement therapy, such as hyaluronic acid (HA) [23]. Artificial tears have multiple functions: they mimic one or more components of natural tears, maintain ocular surface hydration, replenish essential components of the tear film, and provide essential eye lubrication [23]. The changeable formulation of artificial tears is the most significant advantage of this treatment, rendering artificial tears suitable for managing different types of DED [24].

Determining the optimal concentration and combination formulations has been a research area of intense interest in the investigation of artificial tears. Recent surveys revealed that the sequential application of 0.3% and 0.15% HA was superior to either 0.15% or 0.3% HA monotherapy in moderate-to-severe DED patients [25]. In addition to determining the optimal concentration of artificial tears, dual-polymer artificial tear therapy has garnered substantial research interest. According to findings from a 3-month, randomized, controlled, multicenter study, a polymer formulation of carboxymethylcellulose and HA was well tolerated and exhibited superior efficacy to that of carboxymethylcellulose [26]. Moreover, a dual-polymer formulation of hydroxypropyl guar and HA enhanced cellular hydration, barrier reinforcement, and sustained lubrication and enhanced corneal re-epithelialization [27]. These mechanisms mitigate dry eye signs and symptoms in post-cataract surgery patients and improve tear film stability in healthy individuals [27]. Furthermore, the application of nanotechnology increased bioavailability and decreased artificial tear administration times, as discussed in the section on novel delivery systems.

Nevertheless, artificial tears fail to address core pathological mechanisms, such as inflammation or immune dysregulation. Artificial tears offer only short-term symptomatic relief because of their rapid ocular clearance [28]. Additionally, preservatives in artificial tears may induce ocular surface toxicity and conjunctivitis, exacerbating DED. For example, the topical application of benzalkonium chloride, a widely utilized eye drop preservative, successfully induced a DED model characterized by both clinical and histological alterations [29]. Therefore, patients should limit the use of artificial tears with preservatives to 4–6 daily applications [5]. Preservative-free formulations are better tolerated and can be administered more frequently as needed [30]. Innovative physical packaging systems, such as NOVELIA, offer a unique multidose package for preservative-free eye drops [31]. The NOVELIA system can effectively prevent the microbial contamination of preservative-free eye drops for 30 days [31].

##### Biological Blood Products

Biological blood products contain biochemical components analogous to those found in natural tears [32]. However, the susceptibility of these components to degradation compromises their stability during storage [32]. Autologous serum eye drops (ASEDs) are prepared by extracting the patient’s own blood [33]. Compared with artificial tears, ASEDs demonstrate greater safety and therapeutic efficacy in the short-term treatment of DED [33]. Additionally, autologous serum eye drops enhance corneal nerve regeneration and sensory recovery in individuals with ocular graft-versus-host disease-induced DED [34]. Nevertheless, in individuals with anemia or other health issues, acquiring an adequate autologous blood volume from these individuals may not be possible [35]. Allogeneic serum derived from voluntary blood donors alleviates this disadvantage and exhibits efficacy and tolerability similar to autologous serum [35].

Platelet-rich plasma (PRP) drops are autologous preparations of platelets concentrated in a small amount of plasma [36]. Platelets promote the sustained release of growth factors that are responsible for the regeneration of corneal and conjunctival surfaces [36]. Compared with autologous serum, PRP has a shorter preparation time and superior efficacy in decreasing the ocular surface disease index (OSDI) [37]. Nevertheless, preparation protocols, concentrations, and treatment schedules vary markedly across studies, ultimately limiting the clinical application of biological blood products.

##### Omega-3 and Omega-6 Polyunsaturated Fatty Acids

Omega-3 polyunsaturated fatty acids (PUFAs), which are fatty acid supplements, exist in two forms, short-chain (alpha-linolenic acid) and long-chain (eicosapentaenoic acid and docosahexaenoic acid), which are derived from plant-based and marine-based foods, respectively [38]. Two phase II clinical studies demonstrated the clinical efficacy of omega-3 PUFAs for DED; however, significant statistical heterogeneity was also observed among these trials [39,40]. This observed heterogeneity may stem from different dropout rates between the two studies. Specifically, participant attrition was considerably greater in the first study among those receiving a high dose of omega-3 fatty acids (960 mg of docosahexaenoic acid plus 1440 mg of eicosapentaenoic acid daily) [39] than in the second study using a lower dose (480 mg of docosahexaenoic acid plus 720 mg of eicosapentaenoic acid daily) [40], mainly because of more severe gastric intolerance in the high-dose group. Notably, in a multicenter, double-blind phase III clinical trial (NCT02128763), compared with patients in the placebo control group, DED patients who received 3000 mg of omega-3 fatty acid supplements daily for 12 months did not achieve significantly superior clinical outcomes [41]. A recent phase II clinical study also failed to show any beneficial effect of the re-esterified triglyceride form (lower gastrointestinal risk) of omega-3 fatty acids in alleviating the symptoms of MGD-related DED [42]. Although this study was limited by its small sample size (fewer than 60 participants in each group), the efficacy of omega-3 PUFAs warrants further investigation [42].

Omega-6 PUFAs were reported to reduce symptom scores and improve corneal fluorescein staining relative to placebo in an RCT [43]. Nevertheless, two systematic reviews failed to demonstrate the efficacy of omega-6 supplementation alone in managing dry eye disease [38,44].

##### GlicoPro

GlicoPro, a sterile snail mucus extract, enhanced the regeneration and bioadhesivity of corneal cells and exhibited anti-inflammatory and analgesic properties in an in vitro human corneal tissue DED model [45]. Furthermore, a prospective longitudinal clinical study confirmed that 10% GlicoPro significantly attenuated OSDI questionnaire scores and ocular pain in patients with severe DED [46]. However, this investigation was limited by the small cohort of 30 participants. A pilot clinical study also indicated that GlicoPro significantly alleviated ocular discomfort and increased both tear volume and stability; however, the absence of a placebo-controlled group and a small sample of 60 individuals restricts the generalizability of these results [47]. Moreover, another investigation evaluated the combined regimen of GlicoPro and hydroxypropyl-methylcellulose [48]. Both in vitro and clinical studies demonstrated that GlicoPro enhances the therapeutic effect of hydroxypropyl-methylcellulose, potentially through the downregulation of key inflammatory factors, such as IL-1β, TNF-α, and matrix metalloproteinase 9 (MMP-9) [48]. Given this promising yet preliminary evidence, the clinical efficacy of GlicoPro still needs to be defined.

#### 4.1.2. Anti-Inflammatory Drugs and Immunosuppressants

Corticosteroids (e.g., loteprednol) prevent ocular surface inflammation likely by inhibiting NF-κB activation, promoting lymphocyte apoptosis, and reducing the expression of proinflammatory cytokines including IL-1 and TNF-α [49]. In an investigation using rabbit corneal epithelial cells and rabbit models, hydrocortisone was shown to cross the corneal barrier in a dose-dependent manner [50]. At the lowest concentration tested (0.001%), no hydrocortisone was detected in aqueous humor, indicating that the drug did not cross the corneal barrier at this dose [50]. Thus, utilizing a low concentration of hydrocortisone may reduce the side effects of hydrocortisone. A combination of 0.2% hyaluronic acid and 0.001% hydrocortisone sodium phosphate (Idroflog) has been granted approval by the European Union and is commercially available [51]. Nevertheless, the prolonged use of topical corticosteroids is not recommended because of the increased risk of cataract formation, glaucoma, and ocular infection [49]. Notably, nonsteroidal anti-inflammatory drugs (NSAIDs), such as diclofenac sodium, relieve these corticosteroid-induced adverse effects and high drug dependency [49].

Apart from corticosteroids and NSAIDs, tetracycline and macrolide antibiotics, including doxycycline, minocycline, and azithromycin, are also frequently utilized in the management of DED, as well as for the treatment of MGD and blepharitis [7,52]. However, antibiotic-associated adverse effects are frequently observed. Therefore, long-term administration is not recommended.

Immunosuppressants attenuate cytokine secretion and inhibit the activation of effector T lymphocytes. Cyclosporin A (CsA), a neutral lipophilic cyclic undecapeptide derived from fungi, acts as a highly specific inhibitor of T-cell activation [53]. In the ESSENCE-2 randomized clinical trial, compared with vehicle treatment, 0.1% water-free cyclosporine solution produced an earlier therapeutic response in patients with moderate-to-severe DED [54]. In a prospective randomized controlled trial (RCT), treatment with 0.03% tacrolimus, a macrolide antibiotic derived from *Streptomyces tsukubensis*, had better efficacy than treatment with 0.05% CsA [55]. Notably, CsA and tacrolimus are frequently associated with a delayed onset of symptom improvement.

#### 4.1.3. Perfluorohexyloctane

Perfluorohexyloctane (NOV03), a topical, FDA-approved ophthalmic solution for DED, serves as a single-component, preservative-free, and water-free formulation based on semifluorinated alkane technology [56]. NOV03 forms a protective monolayer at the air–tear film interface, thereby diminishing tear evaporation [56]. Clinical data demonstrate improved ocular tolerability, with lower rates of instillation site discomfort for perfluorohexyloctane than for CsA [57]. The therapeutic efficacy of NOV03 was substantiated in two large, controlled phase 3 clinical trials (the MOJAVE study [58] and the GOBI study [59]). Additionally, NOV03 was specifically indicated for MGD-related DED [58,59].

#### 4.1.4. Receptor Agonists

OC-01 (varenicline solution) nasal spray is a highly selective nicotinic acetylcholine receptor (nAChR) agonist approved by the FDA [60]. Its therapeutic mechanism relies on the presence of nAChRs on trigeminal nerve terminals within the nasal cavity. When activated by OC-01, the trigeminal nerve stimulates the lacrimal functional unit to produce tears [61]. Clinical evidence from both phase 2b [62] and phase 3 trials [63,64] demonstrated that OC-01 improved tear film production. The most frequently reported adverse events were sneezing and coughing; these effects were generally mild and occurred within one minute after dosing [65]. Further investigations are needed to identify which DED subtypes are most likely to respond to OC-01 [64].

The P2Y2 receptor, a member of the G protein-coupled receptor family, is prominently expressed in multiple ocular surface tissues, including goblet cells, corneal epithelial cells, and both sebaceous acinar and ductal cells of the meibomian glands [66]. A retrospective observational study demonstrated that diquafosol, a P2Y2 receptor agonist, stimulated tear and mucin secretion, thereby alleviating symptoms in DED patients [67]. Another retrospective observational study demonstrated that diquafosol treatment stimulated the expression of nerve growth factor, subsequently enhancing corneal wound healing [68].

#### 4.1.5. Sex Hormones

The association between sex hormones and DED remains a subject of ongoing debate, with the underlying pathogenic mechanisms yet to be fully elucidated. One proposed mechanism involves the competitive binding of estrogen to androgen receptors, thereby antagonizing the androgen-mediated stimulation of meibomian gland function [69]. A proposed alternative hypothesis is that evaporative DED during menopause may be attributed primarily to a reduction in testosterone levels [70]. In contrast to estrogens, androgens enhance the function of the meibomian glands and suppress the synthesis of IL-1β and TNF-α [71]. Although both estrogen and androgen receptors are expressed in the meibomian glands, androgen receptors are more pivotal in regulating tear secretion [71]. However, in a case–control study of 22 postmenopausal women with severe evaporative DED, serum levels of 17-β-oestradiol, oestrone, and total testosterone were significantly lower in the case group than in the control group [72]. Furthermore, within the case group, levels of 17-β-oestradiol, oestrone, and total testosterone were inversely correlated with tear film osmolarity [72]. These findings indicate that the relationship between sex hormones and dry eye warrants further investigation.

### 4.2. Biological Agents

#### 4.2.1. Mesenchymal Stromal Cells and Their Derivatives

Mesenchymal stromal cells (MSCs) possess anti-inflammatory, tissue-reparative, and immunomodulatory capabilities [73]. Compared with bone marrow-derived MSCs, the MSC population isolated from human lacrimal glands demonstrates comparable phenotypic characteristics, clonogenic potential, and multilineage differentiation capacity [73]. Notably, lacrimal gland-derived MSCs exhibit increased IL-1β secretion and lack the expression of lacrimal epithelial cell markers, which downregulate immunogenicity [74]. In a phase I clinical trial, the administration of allogeneic adipose-derived stem cells into the lacrimal glands improved the OSDI score, tear film osmolarity, tear film breakup time (TBUT), Oxford corneal staining score, and Schirmer’s test values in severe aqueous-deficient dry eye patients [75]. In Sjögren’s syndrome-associated DED, a phase II clinical trial investigated the therapeutic potential of adipose-derived stem cell transplantation in the lacrimal glands [76]. Compared with untreated controls, the intervention group exhibited improved subjective parameters (OSDI score) and objective clinical measures (including noninvasive TBUT, tear meniscus height, Schirmer’s test values, and Oxford staining scores) throughout the 12-month follow-up period [76].

The therapeutic effects of MSCs can be similarly mimicked by MSC-derived exosomes (MSC-Exos), which are purified from MSC-conditioned culture medium and offer distinct advantages as a cell-free alternative [73]. Compared with whole MSCs, MSC-Exos demonstrate reduced immunogenicity and improved stability while eliminating safety concerns linked to viable cellular therapies [73]. In a murine model of DED, MSC-Exos restricted the dendritic cell-dependent immune response, specifically by restraining Th17 polarization [77]. Additionally, MSC-Exos suppressed the production of proinflammatory cytokines (TNF-α, IL-6, and IL-1β), attenuated the Th17 cell population, and impaired dendritic cell recruitment and maturation [77]. In another preclinical murine model, human umbilical cord-derived MSC-Exos modulated the IRAK1/TAB2/NF-κB signaling cascade, facilitated by specific microRNAs, such as miR-125b, let-7b, and miR-6873 [78]. In addition to these miRNAs, miR-146a overexpression enhanced human corneal epithelial cell viability while suppressing apoptotic activity and inflammatory responses in vitro and in murine DED models [79]. The upregulation of SQSTM1 expression by miR-146a may promote cell survival and attenuate apoptosis and inflammation in human corneal epithelial cells [79].

#### 4.2.2. Fibroblast Growth Factor 10

Fibroblast growth factor 10 (FGF10) is selectively enriched in mesenchymal cells and activates the paracrine activity of FGF receptor 2b on neighboring epithelial cells [80]. This ligand–receptor system critically regulates developmental and homeostatic processes in ocular surface tissues, including meibomian gland morphogenesis and corneal epithelial integrity [80]. In a rabbit dry eye model, exogenous FGF10 supplementation increased mucin biosynthesis in the conjunctival epithelia [81]. Moreover, SLC7A11 is involved in the protective effects of FGF10 against oxidative stress, endoplasmic reticulum stress, and apoptosis in both murine corneal epithelium and human HCE-2 cells [82]. Thus, investigations on FGF10 remain at an early, preclinical stage.

### 4.3. Novel Drug Delivery Systems

Conventional drug administration typically mitigates systemic side effects by decreasing drug absorption [83]. However, for tear substitute medications such as artificial tears, the use of traditional administration routes may disrupt the natural microenvironment of the ocular surface [83]. The topical application of ophthalmic solutions may induce ocular irritation and reflex blinking responses, thereby accelerating drug dilution [84]. Under physiological conditions (isoelectric point ~3.2), the corneal epithelium is anionic and preferentially permeable to cationic therapeutic agents [84].

Compared with conventional interventions, nanoparticle-based ocular drug delivery systems can prolong precorneal retention, increase ocular drug bioavailability, and maintain therapeutic efficacy with minimal side effects [85]. For instance, dexamethasone-loaded cationic nanostructured lipid carriers functionalized with a chondroitin sulfate-L-cysteine conjugate provide possible strategies for overcoming the ocular biological barrier [86]. In this complex, L-cysteine is conjugated to chondroitin sulfate, a natural glycosaminoglycan in extracellular matrices and cellular surfaces, to confer anti-inflammatory properties and preserve tissue integrity [87]. Moreover, the introduction of cationic moieties enhances electrostatic interactions with the negatively charged mucosal layer [88]. In a rabbit model of DED, this complex enhanced corneal epithelial restoration and tear film stabilization and alleviated ocular symptoms without detectable adverse effects [86]. Another example of a nanoparticle, consisting of catalase self-assembled with thiolated chitosan for ocular delivery, exhibited superior therapeutic efficacy compared with conventional cyclosporin and dexamethasone treatment in mouse and rabbit models [89]. This innovative formulation capitalizes on the unique properties of chitosan, a natural biodegradable cationic polymer with an intrinsic ability to adhere to the mucosa [90]. The thiolation process employs cysteine, a compound designated by the FDA as generally safe, to modify chitosan [91]. This system forms disulfide bonds with cysteine-rich mucin glycoproteins in the tear film, thereby enhancing ocular surface retention [89].

CsA-based nanoemulsions (Restasis [92] and Cyclokat [93]) and nanocolloid formulations (OTX-101 [94]) have been approved for clinical use and are commercially available. Compared with CsA-based nanoemulsions, CsA lipid nanocapsules similarly ameliorated ocular targeting, exhibited sustained drug release, and demonstrated optimized biocompatibility in a dry eye rabbit model [95].

Nanotechnology can integrate diverse functional components to achieve synergistic functions. For instance, Li et al. engineered cationic polypeptide micelles coloaded with losmapimod and Tempo to synergistically target oxidative stress and inflammation [96]. Losmapimod, a clinically well-tolerated selective p38 mitogen-activated protein kinase (MAPK) inhibitor, effectively suppresses inflammatory cytokine production mediated by the p38 MAPK signaling pathway [97]. Tempo, a cost-effective and stable nitroxide radical, exhibits potent superoxide dismutase-mimetic antioxidant activity [98]. These cationic micelles increase ocular bioavailability by prolonging corneal retention through electrostatic interactions with negatively charged mucin. In a mouse model, this dual-action micelle system attenuated inflammatory cascades, promoted corneal epithelial repair, preserved goblet cell function, and restored tear secretion [96]. A preclinical study suggested that ferroptosis may contribute to the pathogenesis of DED [99]. A sialic acid-targeting peptide-modified liposomal delivery system with coencapsulated CsA and ferrostatin-1, a selective ferroptosis inhibitor, was developed [100]. This formulation exhibited enhanced aqueous solubility and prolonged ocular surface retention in a murine model [100].

Another potential benefit of nanoparticles is that they can simultaneously deliver both lipophilic and hydrophilic agents. For instance, HA exhibits remarkable water-binding capacity and minimal immunogenicity [101]. Nevertheless, the clinical application of HA-based ophthalmic formulations faces critical limitations, including rapid precorneal clearance caused by its hydrophilicity and the physiological blinking reflex [101]. Inspiringly, HA-based conjugates with precisely controlled oleic acid substitution represent a novel nanomaterial for enhanced ocular codelivery of CsA and oleic acid [102]. Another HA-coated liposomal formulation encapsulates lactoferrin, a multifunctional iron-binding glycoprotein. This formulation demonstrated stronger anti-inflammatory efficacy than free lactoferrin in both in vitro experiments and in vivo rabbit models [103].

### 4.4. Device-Based Therapies

#### 4.4.1. Photobiomodulation Therapies

Current photobiomodulatory therapies for DED primarily include intense pulsed light therapy (IPLT) and low-level light therapy (LLLT). IPLT induces selective photothermolysis in periorbital tissues via sequential noncoherent, broad-spectrum light pulses [104]. The potential therapeutic mechanisms involve anti-inflammatory cytokine modulation, elimination of *Demodex*, and photothermolysis targeting abnormal periocular subdermal blood vessels [105].

In a retrospective case series, the therapeutic efficacy of IPLT in 110 participants with MGD-related DED was evaluated over a 12-month follow-up period [106]. The results revealed elevated subjective indicators (eye fatigue scores) and objective parameters (tear film stability and inflammatory markers) [106]. In a phase II clinical trial, IPLT intervention significantly improved various ocular surface parameters, including the TBUT, tear film lipid layer thickness, and meibomian gland quality, in severe evaporative DED patients [107]. However, the study’s statistical power may be limited by the small cohort size (49 adult participants) [107].

LLLT refers to the application of lasers or specific light sources at low photon energy densities to induce photobiomodulation without thermal effects [108]. Compared with IPLT (3000 K, 4000 K and 6600 K), LLLT (1900 K) demonstrated greater clinical benefits, including increased melatonin and glutamate secretion, ocular protection, and accelerated wound healing [109]. This therapeutic approach transfers photon energy to cellular chromophores, initiating photochemical reactions [108,109]. In both rabbit and rat models of DED, LLLT treatment resulted in enhanced tear synthesis, reduced fluorescein staining scores, decreased inflammatory mediator levels, and decreased corneal and conjunctival epithelial apoptosis [110]. In DED patients, the administration of LLLT also has multiple therapeutic effects, including prolonging the TBUT, increasing the meibomian gland height and tear film lipid layer thickness, and enhancing tear secretion while simultaneously decreasing corneal staining scores and Schirmer’s test results [110].

The underlying mechanism of LLLT also remains to be fully elucidated but may involve enhanced mitochondrial ATP synthesis via photostimulation, as demonstrated in animal and in vitro experiments [111]. Furthermore, preclinical studies revealed that light irradiation may facilitate the photodissociation of nitric oxide from cytochrome c oxidase, thereby enhancing oxidative phosphorylation by reversing nitric oxide-mediated respiratory inhibition [112]. This photochemical process also generates reactive oxygen species (ROS) that are below risk levels and function as signaling molecules to modulate multiple cellular pathways [112].

A novel low-level light therapy device (my-mask, Espansione Marketing S.p.A., Bologna, Italy) is available for home use and has demonstrated superior clinical efficacy compared with artificial tears and eyelid hygiene in managing MGD-related DED [113]. Moreover, in a prospective RCT, two sessions of LLLT, administered preoperatively (7 ± 2 days before cataract surgery) and postoperatively (7 ± 2 days after cataract surgery) for the prevention of postoperative DED, markedly enhanced tear film stability and relieved ocular discomfort [114].

The integration of nanotechnology and photobiomodulation therapy has yielded innovative therapeutic strategies [115]. Compared with monometallic palladium or gold nanoparticles at equivalent concentrations, palladium-coated gold nanoparticles exhibited superior photothermal conversion efficiency under visible light irradiation, effectively stimulating lacrimal gland secretion [116]. Pang et al. utilized palladium-coated gold nanoparticles to construct a photothermal conversion hydrogel-based mini-eye patch, which was pasted to the lacrimal gland [115]. This patch significantly improved tear film stability, as shown by a prolonged TBUT and increased tear meniscus height [115]. Compared with traditional infrared goggles, the proposed mini-eye patch is a more user-friendly therapeutic option.

#### 4.4.2. Thermal Pulsation Therapies

The LipiFlow system employs a dual-mechanism approach to treat MGD. It utilizes targeted heating to melt the obstructed meibum on the inner eyelid surface, concurrently applying rhythmic external eyelid compression to facilitate its expulsion [117]. Specifically, by heating the conjunctival eyelid surface to 41–43 °C, the LipiFlow system targets the pathologically elevated melting point of the meibum in MGD (the normal range of 32–40 °C) to liquefy stagnant secretions [117]. A clinical trial review spanning 15 years revealed that a single 12 min LipiFlow treatment safely and effectively improved the manifestations of MGD and associated evaporative DED [118]. The resulting clinical benefits persisted for up to 3 years in some cases, and the procedure did not cause accompanying discomfort or pain [118]. Nevertheless, most of the currently available evidence has a high risk of bias, resulting in low certainty [119]. Consequently, more rigorous studies—incorporating adequate blinding, standardized diagnostic protocols, and cohorts representative of the broader MGD population—are needed to support its clinical application [119].

The iLUX system, a handheld, battery-powered instrument, operates on the same fundamental principle as the LipiFlow system [120]. Notably, the iLUX system incorporates a built-in magnifying lens, which allows for precise localization of obstructed meibomian gland orifices [121]. This functionality facilitates manual, real-time adjustment of both temperature and compressive pressure [121].

#### 4.4.3. Neurostimulation Therapies

##### Intranasal Tear Neurostimulation

The intranasal tear neurostimulation device activates the nasolacrimal reflex by delivering microcurrents to the trigeminal nerve, thereby enhancing basal tear secretion. A recent meta-analysis of the efficacy of intranasal tear neurostimulation for the treatment of DED revealed a significant increase in Schirmer II test scores and the meibomian gland area, accompanied by mild-to-moderate adverse events but no serious adverse effects [122]. Furthermore, in individuals with DED, intranasal neurostimulation increased tear production and concurrently alleviated the severity of dryness and ocular pain [123]. Future investigations should employ varying current intensities and frequencies to further validate its clinical efficacy [122].

##### Transcutaneous Electrical Nerve Stimulation

Transcutaneous electrical nerve stimulation (TENS), a noninvasive neuromodulation technique, delivers electrical currents via surface electrodes to directly activate peripheral neural pathways and elicit secondary effects within the central nervous system through established neural connections [124]. Periorbital electrical stimulation increases the blink frequency and the elicitation of forceful blink reflexes by stimulating motor nerve axons and sensory fibers. Extended interblink periods can result in elevated tear osmolarity, which may contribute to inflammatory responses [125]. Consequently, periorbital electrical stimulation increases tear film stability. This effect may be mediated by the potentiated contractions of periorbital muscles induced by TENS, thereby facilitating meibomian gland secretion. In a clinical study involving healthy users of video display terminals, a series of six 30 min sessions of periorbital electrical stimulation significantly alleviated ocular discomfort in individuals with DED [126]. This therapeutic intervention effectively alleviated ocular discomfort and increased both tear secretion and tear film stability through the induction of robust blink reflexes without interfering with visual function [126]. However, the long-term therapeutic benefits and potential for clinical implementation in patients warrant further comprehensive investigation.

##### Quantum Molecular Resonance

Quantum molecular resonance (QMR) electrical stimulation employs a low-intensity, high-frequency current via electromagnetic patches on the skin near the lower eyelid and close to cranial nerves V1 and V2. This modality induces a cyclic process of contraction and relaxation in targeted cells, which promotes cellular metabolic activity, facilitates tissue regeneration, and improves structural and functional integrity. Furthermore, QMR downregulates the expression of MMPs and attenuates leukocyte infiltration, contributing to the anti-inflammatory properties of this technique [127,128]. Electrical signals transmitted to the trigeminal nerve and lacrimal system stimulate the lacrimal and meibomian glands, thereby enhancing tear secretion and increasing the thickness of both the lipid and mucin layers [129]. In an early clinical study, QMR therapy improved inferior corneal staining and enhanced subjective dry eye symptom scores, with no adverse events reported [127]. A phase II clinical study demonstrated that compared with tear substitutes containing 0.15% HA and 3% trehalose, QMR was a well-tolerated and more effective therapeutic option for patients with severe DED, with significant improvements in subjective and objective ocular parameters [128]. This efficacy was consistently observed across other DED subtypes, including mild, aqueous-deficient, evaporative, and mixed-type dry eye, in two phase IIa clinical studies [129,130].

##### Transcranial Magnetic Stimulation

Transcranial magnetic stimulation (TMS) mainly alleviates the symptoms of DED induced by lacrimal gland impairment [131]. In a clinical investigation involving moderate-to-severe dry eye of mixed etiologies, each participant received a series of 32 TMS pulses, with the stimulation intensity progressively increasing to 45% of the maximum output. Subsequent evaluations demonstrated elevated corneal staining scores and relief of dry eye-related symptoms [131,132]. In a preliminary clinical study, repetitive magnetic stimulation improved tear film stability and supported corneal health without adversely affecting intraocular pressure, visual acuity, or tear secretion [132]. Nevertheless, the precise mechanism underlying TMS remains to be fully elucidated and warrants further investigation.

##### Sonic Stimulation

Stimulation of the external nasal nerve using sonic vibration can activate the lacrimal functional unit [133]. The iTEAR100 device (Olympic Ophthalmics), a novel portable instrument for external sonic neuromodulation, is equipped with a unidirectional oscillating tip operating at a frequency of approximately 220–270 Hz and an amplitude of approximately 0.5–1 mm. The oscillating tip is positioned at the junction between the nasal cartilage and bone on both sides of the nose, and each side is stimulated for 30 s per day over at least two treatment sessions [134]. In a pivotal phase III clinical study, patients exhibited significant improvements in OSDI, lacrimal secretion test results, and corneal and conjunctival staining scores during the 30-day trial period [134]. The improvement in lacrimal secretion and OSDI scores was still observable at the 180-day follow-up. Three mild adverse events, namely, transient headache, sneezing, and episodic nasal pain, were found to be related to the device [134].

#### 4.4.4. Acupuncture

Acupuncture, a nonpharmacologic traditional Chinese medicine approach [135], can improve the TBUT, Schirmer test results, corneal fluorescein staining, and symptom scores [136,137]. A systematic review and meta-analysis revealed that an optimal treatment regimen for typical dry eye syndrome is acupuncture administered 2–3 times weekly for 21–30 days [136]. Additionally, a prospective sham-controlled phase II clinical trial demonstrated that acupuncture improved mainly the Schirmer test results, corneal fluorescein staining score and TBUT [137]. In another phase IIa clinical study, acupuncture ameliorated Sjögren’s syndrome through a multifaceted mechanism involving the upregulation of AQP1 and AQP5 expression, the suppression of proinflammatory cytokines (IL-17 and TNF-α), and the subsequent attenuation of glandular inflammation [138]. Nevertheless, recent studies are limited by small sample sizes.

### 4.5. Surgical Treatments

**Figure 3:**
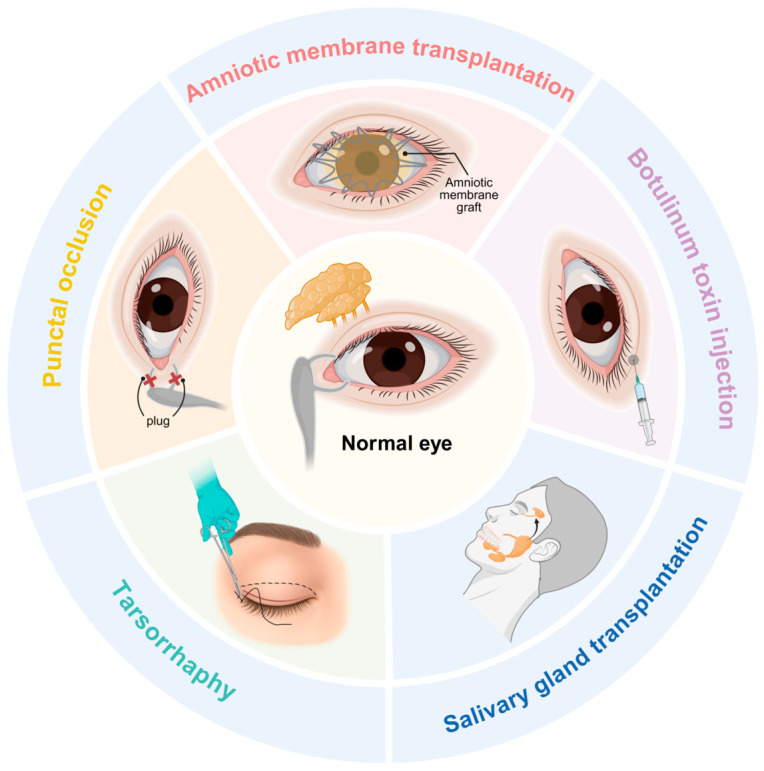
Surgical approaches. Amniotic membrane transplantation involves surgical placement of the amniotic membrane onto the ocular surface. Botulinum toxin injection therapy involves the injection of neurotoxin into the lacrimal gland. Conjunctival laxity excision surgically removes redundant lower bulbar conjunctival tissue. Tarsorrhaphy procedures involve specialized suturing techniques to minimize palpebral fissure width and decrease ocular surface exposure. Punctal occlusion therapy involves the use of silicone or collagen plugs inserted into the lacrimal puncta to physically block tear drainage.

Surgical approaches are typically applicable in refractory cases that are unresponsive to conventional pharmacological therapies. The five main surgical interventions for DED are depicted in Figure 3. Tarsorrhaphy achieves partial or complete eyelid closure through either temporary or permanent means. The primary objective of this intervention is to minimize ocular surface exposure, thereby attenuating tear film evaporation and ultimately alleviating ocular surface desiccation [139]. Botulinum toxin A (BTX-A) potentiates tear secretion through the presynaptic inhibition of acetylcholine release at neuromuscular junctions, inducing temporary muscle paralysis [140]. Nevertheless, Sawaed et al. demonstrated that BTX-A administration disrupted meibomian gland function, resulting in a significantly decreased TBUT and exacerbated DED. The limitation of this investigation was the small sample size (26 patients) [141]. Punctal occlusion preserves tear film stability on the ocular surface through the insertion of punctal plugs in one or both puncta to mechanically obstruct tear drainage [142]. Punctal plugs are categorized into temporary/absorbable types (typically collagen-based) and nonabsorbable/permanent types (typically silicone-based) [142]. Surgical punctal occlusion combined with canalicular ablation and punctal suturing can attenuate the corneal staining score, Schirmer tear test score, and subjective symptoms, effectively alleviating the high recanalization rate in individuals with severe DED [143]. Nevertheless, punctal plugs are not suitable for all DED patients. In DED patients with active ocular surface inflammation or poor tear quality, tear retention may prolong the contact time of inflammatory mediators, debris, or toxic tear components with the ocular surface, potentially exacerbating symptoms.

Amniotic membrane transplantation is an emerging and multifunctional surgical therapeutic approach that facilitates ocular surface reconstruction, repairs conjunctival defects, manages limbal stem cell deficiency, and promotes tissue regeneration [144,145]. In particular, this surgery is readily performed at the bedside [145]. The amniotic membrane, the innermost placental layer enveloping the developing fetus, is composed of a monolayer of epithelial cells, a dense basement membrane, and an avascular stromal compartment rich in extracellular matrix components [144]. Yi et al. recently revealed that human amniotic epithelial cells–extracellular vesicles (hAEC-EVs) play a role in the therapeutic effects of the amniotic membrane [146]. In this study, hAEC-EVs not only enhanced human corneal epithelial cell proliferation and migration capacity but also effectively suppressed inflammatory cytokine production in vitro. In a DED mouse model, hAEC-EVs attenuated corneal staining scores and restored tear secretion, corneal surface regularity, and conjunctival goblet cell density [146].

Salivary gland transplantation, which involves mainly autologous transplantation of the submandibular gland to the temporal fossa (SMGT) and minor salivary gland transplantation (MSGT), is a promising surgical treatment for similar acinar–ductal structures [147]. With duct placement into the conjunctival fornix, SMGT markedly increases tear volume, although the synthesis of a hypoosmotic tear film may lead to corneal edema [148]. MSGT performed in the fornix provides a smaller improvement in Schirmer values but is associated with a lower risk of reflex epiphora than SMGT [147]. Notably, following corneal transplantation, visual acuity and corneal graft outcomes exhibit considerable variability, highlighting the need for further investigations.

### 4.6. Lifestyle and Environmental Interventions for DED

Exposure to low-humidity conditions (e.g., air-conditioned or windy environments) increases the vapor pressure gradient at the ocular surface, leading to accelerated tear evaporation [2]. A 10% decrease in relative humidity can increase the evaporation rate by 28–59% [2]. While increased workplace illumination reduces the risk of tear film instability, the low air humidity and use of air conditioning characteristic of office environments increase the risk of ocular dryness and intensify the symptoms of digital eye strain [149].

Screen-associated dry eye is caused by multiple etiologies, including blink abnormalities, meibomian gland and goblet cell dysfunction, and the photobiological impact of peak emission wavelengths from modern LEDs on the cornea [150]. To prevent screen-associated dry eye, a multimodal approach encompassing blink retraining, omega-3 supplementation, and ergonomic optimization targets distinct pathophysiological mechanisms, allowing for complementary and potentially synergistic effects [151].

Dietary intake, including the intake of multiple vitamins and caffeine, represents another modifiable and beneficial lifestyle factor. Vitamin D regulates the cell cycle to strengthen the corneal epithelial barrier and modulates systemic calcium absorption to maintain aqueous and lipid secretion [152]. In addition, vitamin D insufficiency is correlated with the pathogenesis and severity of dry eye [152]. Vitamin C, an antioxidant, can alleviate oxidative stress on the ocular surface and aid in corneal wound healing in DED patients [153]. Vitamin A is essential for maintaining the homeostasis of ocular mucosal tissues and facilitating phototransduction in the retina [154]. Specifically, vitamin A contributes to the metabolic activity, growth, and differentiation of ocular surface epithelial tissues [154]. Vitamin B12 effectively relieves DED-related neuropathic ocular pain, especially in severe cases that are unresponsive to topical treatments [155]. Moreover, a cross-sectional analysis elucidated the potential protective role of caffeine in dry eye [150].

Psychological factors are considered emerging contributing factors to the pathogenesis of DED. Multivariate regression analysis revealed an inverse association between improved self-perceived health status and the incidence of DED [156]. Conversely, increased self-reported psychological stress was positively associated with all three ocular conditions [156]. Furthermore, DED has a complex bidirectional relationship with psychological disorders, particularly depression and anxiety, thereby forming a vicious cycle: psychological distress exacerbates DED symptoms, which in turn further compromises patient quality of life [157].

## 5. Discussion and Future Perspectives

**Figure 4:**
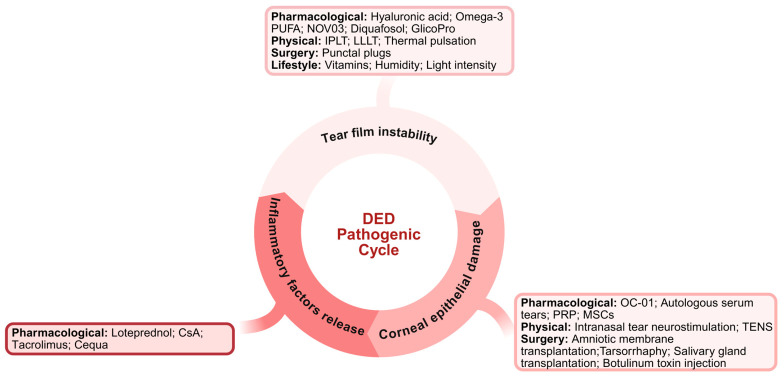
Schematic summary integrating pharmacological, procedural, and lifestyle interventions for the pathogenesis of DED. The position of the therapies in the figure is based on their primary mechanism.

In this review, we critically analyze and evaluate the molecular mechanisms and clinical efficacy of both classic and emerging management strategies for DED. The TFOS DEWS III categories, DED subtypes, main indications, evidence levels and limitations of the primary interventions for DED are described in Table 1. Pharmacological, procedural, and lifestyle interventions for the pathogenesis of DED are summarized in Figure 4. Currently, the limitations of conventional pharmacological therapies, such as rapid clearance, the adverse effects of preservatives, and the targeting of symptoms rather than etiologies, have spurred research interest in alternative approaches, such as photobiomodulation therapy, nanoparticle-based drug delivery systems, biological agents and neurostimulation. Although surgical interventions often provide longer-lasting effects than pharmacological treatments do, their application is limited by potential postoperative complications and the complexity of the surgical procedures. Additionally, benign lifestyle interventions promote the establishment of a comprehensive and patient-centered therapeutic paradigm.

However, contemporary therapeutic innovations encounter substantial challenges in their clinical translation. The efficacy and duration of photobiomodulation and thermal pulsation therapy vary significantly on the basis of multiple factors, such as the light intensity/temperature, the number of treatment sessions, differences in instrumentation, and the algorithms employed. The development of nanotechnology is routinely impeded by the need for multistage fabrication workflows that yield batch-to-batch variability and colloidal dispersion instability [162]. Minor variations in specific process parameters can trigger pronounced fluctuations in particle size and product yield. These critical parameters directly govern encapsulation efficiency, drug release kinetics, and overall therapeutic efficacy. Such batch-to-batch variability may further alter the pharmacokinetic profiles and pharmacological properties of active pharmaceutical ingredients, ultimately complicating quality control [163]. Thus, poor reproducibility limits manufacturing scalability, impeding the clinical use of nanocarriers. Additionally, evidence concerning the biocompatibility, toxicity, and metabolic fate of nanoparticles within the eye remains insufficient [164]. In addition, nanocarriers exhibit unique physicochemical properties because of their particle size, stressing the importance of specialized regulatory guidelines. Regulatory guidelines for nanomedicine products are being developed by diverse regulatory bodies (including the US FDA and EMA), yet such frameworks remain in their infancy [165]. With respect to MSCs, their limited survival time and the implantation of bioactive agents represent critical challenges in the wide application of MSCs for DED. Furthermore, clinical trials involving MSCs predominantly focused on patients with severe autoimmune dry eye disease, as defined by an OSDI score ≥33 and Schirmer’s test results of 1–5 mm/5 min [76]. Additionally, studies of amniotic membrane transplantation have been limited primarily to general RCTs [144]. Therefore, independent and large-scale RCTs are needed to determine various aspects of innovative therapies, such as clinical validity, side effects and appropriate dosage.

Future investigations should prioritize the development of multimodal therapeutic approaches, particularly nanoparticle-based delivery systems. Subsequent research efforts should focus on optimizing the biodegradability and economic viability of nanocarriers while systematically elucidating their degradation kinetics and drug release profiles in ocular tissues. Moreover, current therapeutic strategies predominantly employ standardized dosing regimens targeting broad patient populations. Personalized therapeutic regimens that are tailored to an individual’s genetic profile, environmental exposures, and lifestyle factors are essential for optimizing drug efficacy and minimizing adverse effects. Future photobiomodulation and thermal pulsation therapies featuring adjustable light parameters may provide a versatile and household-based approach for personalized management. A patient-centric diagnostic approach utilizing integrated metrics such as the Pentascore (which combines five visual analog scales to evaluate ocular discomfort severity, DED-related functional impairment, and therapeutic efficacy/tolerability) allows for swift, holistic assessments of DED burden and patient treatment satisfaction [166]. This strategy facilitates individualized management that accounts for both symptomatic impact and lifestyle-related challenges.

Biomarkers also propel early-phase diagnosis and precision medicine development. For example, MMP-9 serves as an inflammatory biomarker that is upregulated in the tears of patients with DED [167]. In a prospective, sequential, masked, multicenter clinical trial, InflammaDry—a rapid point-of-care assay designed to detect elevated MMP-9 levels—was evaluated in 206 patients and exhibited high sensitivity and specificity [168]. Therefore, the application of InflammaDry can facilitate the early-phase recognition of dry eye. Recently, a cross-sectional study revealed that MMP-3 levels in the tears of DED patients were markedly increased [169]. In addition, the MMP-3 levels in tears were correlated with corneal fluorescein staining scores, suggesting that MMP-3 is a potential biomarker for DED [169].

In addition to biomarkers, artificial intelligence has been applied to drive the automatic classification of images (including interferometry, meibography, and slit-lamp) and the prediction of medical outcomes [170]. Moreover, machine learning approaches promote consistent diagnosis and stratification of severity [170]. Furthermore, a deep learning-enabled efficacy prediction system identifies potential drug candidates by leveraging changes in the gene expression profiles of patients as input [171]. This has propelled the development of drug repurposing, which is defined as the application of a drug originally approved for one disease in the management of an alternative disease [172]. Compared with traditional drug development, drug repurposing offers a considerable advantage, given that all preclinical work has already been conducted [172].

Moreover, the role of effective clinician-patient communication in DED management promotes patient adherence to lifestyle modifications and fosters the establishment of a robust therapeutic alliance. DED should be regarded as not only a collection of clinical signs but also a chronic condition that decreases quality of life and necessitates empathetic and patient-centered management. DED significantly compromises daily activities such as reading and driving and imposes a substantial economic burden through direct medical costs and reduced productivity [157].

Empathy, effective dialog, and active patient engagement are fundamental to building a strong therapeutic alliance in DED care [157]. Recent consensus reviews stress that clear communication is essential for sustaining long-term tear film homeostasis and achieving therapeutic success [173]. A registry data analysis revealed a marked disconnect between patient-reported symptoms and observable clinical signs, particularly among individuals with mixed pathophysiology or neuropathic features [174]. A survey involving 706 DED patients revealed that 31% perceived their condition as a “disease” or even a “handicap”, whereas 66% regarded it as a “discomfort” [175]. A more negative perception of DED was associated with a greater impact on quality of life, along with factors such as delayed diagnosis, consultations with multiple healthcare professionals prior to diagnosis, and the frequent use of treatments [175]. Thus, it is essential for eye care practitioners to establish realistic patient expectations during DED management, which can help patients better understand the chronic nature of this condition and recognize the need for sustained therapy.

## 6. Conclusions

The evolving understanding of DED pathophysiology has prompted treatment advances. Numerous novel therapeutic strategies have been developed to overcome the limitations of conventional approaches. However, the clinical efficacy, appropriate dosing, and adverse effects of most next-generation therapies remain elusive because large-scale clinical trials of these therapies are lacking. Future investigations should focus on tailoring therapeutic strategies based on precision medicine and optimizing clinician–patient consultations to maximize patient compliance.

## Figures and Tables

**Table 1:** Summary of the main interventions for DED mentioned in this article. Therapies are arranged according to the TFOS DEWS III framework.

Therapy	TFOS DEWS III Category	DED Subtype	Main Indications	Evidence Level	Limitation
Hyaluronic acid [23]	Tear supplements and stabilizers	Tear film deficiencies	Aqueous deficiency	Approved	Preservatives
Omega-3 PUFA [39,40]	Tear supplements and stabilizers	Tear film deficiencies	Lipid deficiency	Phase II clinical trial	Gastric intolerance
NOV03 [58,59]	Tear supplements and stabilizers	Tear film deficiencies	Lipid deficiency	Approved	-
Vitamin A [158]	Tear supplements and stabilizers	Tear film deficiencies	-	Phase II clinical trial	-
Punctal plugs [159]	Tear conservation devices	Tear film deficiencies	Aqueous deficiency	RCT	Infection
Diquafosol [67]	Restoration or stimulation of aqueous	Tear film deficiencies	Aqueous/mucin deficiency	Observational study	-
Thermal pulsation (LipiFlow, iLUX) [118,121]	Restoration or stimulation of lipid	Tear film deficiencies	Lipid deficiency	Approved	Burn
IPLT [107]	Restoration or stimulation of lipid	Tear film deficiencies	Lipid deficiency	Phase II clinical trial	Burn
LLLT [113]	Restoration or stimulation of lipid	Tear film deficiencies	Lipid deficiency	RCT	-
Intranasal tear neurostimulation [160]	Neuromodulation/neurostimulation	Ocular surface abnormalities	Neural dysfunction	Approved	-
TENS [126]	Neuromodulation/neurostimulation	Ocular surface abnormalities	Neural dysfunction	RCT	-
OC-01/Varenicline [60]	Neuromodulation/neurostimulation	Ocular surface abnormalities	Neural dysfunction	Approved	-
Loteprednol [49]	Corticosteroids	Ocular surface abnormalities	Inflammation	Approved	Infection
CsA [54]	T-cell immunomodulatory topical drugs	Ocular surface abnormalities	Inflammation	Approved	Delayed onset
Tacrolimus [55]	T-cell immunomodulatory topical drugs	Ocular surface abnormalities	Inflammation	RCT	Delayed onset
OTX-101 [94]	T-cell immunomodulatory topical drugs	Tear film deficiencies	Aqueous deficiency	Approved	-
Autologous serum tears [33]	Ocular surface promotors/regenerators	Ocular surface abnormalities	Epithelial damage	Single arm clinical trial	Susceptibility to degradation
PRP [36]	Ocular surface promotors/regenerators	Ocular surface abnormalities	Epithelial damage	RCT	-
Amniotic membrane transplantation [144]	Ocular surface promotors/regenerators	Ocular surface abnormalities	Epithelial damage	RCT	Surgical complications
Tarsorrhaphy [161]	Surgery	Eyelid-related anomalies	Blink dynamics/lid closure	Observational study	Surgical complications
Salivary gland transplantation [148]	Surgery	Eyelid-related anomalies	Anatomical misalignment	RCT	Surgical complications
Botulinum toxin injection [141]	Surgery	Eyelid-related anomalies	Blink dynamics/lid closure	Cohort study	Surgical complications
GlicoPro [47]	-	Tear film deficiencies	Mucin deficiency	Pilot study	-
MSCs [76]	-	Ocular surface abnormalities	Inflammation; epithelial damage	Phase II clinical trial	Immunogenicity
MSC-Exos [77]	-	Ocular surface abnormalities	Inflammation; epithelial damage	Preclinical	Difficult purification
Cyclokat [93]	-	Ocular surface abnormalities	Inflammation	Approved	-
FGF10 [81]	-	Tear film deficiencies/ocular surface abnormalities	Mucin deficiency; Inflammation; epithelial damage	Preclinical	-

## Data Availability

No new data were created or analyzed in this study.

## Abbreviations

ASEDs	Autologous serum eye drops
BTX-A	Botulinum toxin A
CsA	Cyclosporin A
DED	Dry eye disease
FGF10	Fibroblast growth factor 10
HA	Hyaluronic acid
hAEC-EVs	Human amniotic epithelial cells–extracellular vesicles
IL	Interleukin
IPLT	Intense pulsed light therapy
LLLT	Low-level light therapy
MAPK	Mitogen-activated protein kinases
MGD	Meibomian gland dysfunction
MMP	Matrix metalloproteinase
MSCs	Mesenchymal stromal cells
MSC-Exos	MSC-derived exosomes
MSGT	Minor salivary gland transplantation
nAChR	Nicotinic acetylcholine receptor
NSAID	Nonsteroidal anti-inflammatory drug
OSDI	Ocular surface disease index
PRP	Platelet-rich plasma
PUFA	Polyunsaturated fatty acids
QMR	Quantum molecular resonance
RCT	Randomized controlled trial
ROS	Reactive oxygen species
SMGT	Submandibular gland to the temporal fossa
TBUT	Tear film breakup time
TMS	Transcranial magnetic stimulation
TNF-α	Tumor necrosis factor-α
